# CISA: Contig Integrator for Sequence Assembly of Bacterial Genomes

**DOI:** 10.1371/journal.pone.0060843

**Published:** 2013-03-28

**Authors:** Shin-Hung Lin, Yu-Chieh Liao

**Affiliations:** Division of Biostatistics and Bioinformatics, Institute of Population Health Sciences, National Health Research Institutes, Zhunan, Taiwan; The Roslin Institute, University of Edinburgh, United Kingdom

## Abstract

A plethora of algorithmic assemblers have been proposed for the *de novo* assembly of genomes, however, no individual assembler guarantees the optimal assembly for diverse species. Optimizing various parameters in an assembler is often performed in order to generate the most optimal assembly. However, few efforts have been pursued to take advantage of multiple assemblies to yield an assembly of high accuracy. In this study, we employ various state-of-the-art assemblers to generate different sets of contigs for bacterial genomes. A tool, named CISA, has been developed to integrate the assemblies into a hybrid set of contigs, resulting in assemblies of superior contiguity and accuracy, compared with the assemblies generated by the state-of-the-art assemblers and the hybrid assemblies merged by existing tools. This tool is implemented in Python and requires MUMmer and BLAST+ to be installed on the local machine. The source code of CISA and examples of its use are available at http://sb.nhri.org.tw/CISA/.

## Introduction

Recently, technological advances have dramatically improved throughput and quality of next-generation sequencing (NGS), and, in parallel with these improvements, numerous algorithms have been proposed for *de novo* sequence assembly. Compared to the traditional Sanger sequencing technology, NGS technologies offer several distinct features, such as large volumes of reads and concise length. In order to tackle the sequence assembly problem from a collection of short-sequencing reads of randomly sampled fragments, two types of algorithm, the overlap-layout-consensus approach and the de Bruijn graph, are commonly utilized [1], [2]. Although assemblers are generally based on a small number of algorithms, they differ from each other in terms of dealing with errors, inconsistencies, and ambiguities. Moreover, no individual assembler guarantees the best assembly for diverse species. Therefore, the use of various parameter settings and assemblers in an iterative manner to produce an improved draft assembly is inevitable. Nevertheless, few efforts have been made to integrate various assemblies into a draft that is marked by superior contiguity and accuracy. To the best of our knowledge, five tools, including GAA [3], GAM [4], MAIA [5], minimus2 [6] and Reconciliator [7], have been published for merging assemblies. However, GAM and Reconciliator require the original reads and complicated prerequisites; we therefore compared our proposed method with GAA, MAIA and minimus2.

The quality of genome assemblies is usually evaluated by means of their contiguity and the accuracy of contigs or scaffolds. Contiguity is a straightforward measurement achieved by calculating the N50 length or the number of contigs/scaffolds, without mandating reference genome information to be implemented in the algorithm. On the other hand, the accuracy of an assembly can be assessed based on alignment with a complete reference genome [8]. However, there is an inevitable trade-off between contiguity and accuracy in such scenarios. Specifically, an assembler attempting to maximize contiguity might do so at the expense of a less accurate assembly, and vice versa. Since an individual assembler has its own features in addressing the reconstruction of a DNA sequence, can we take advantage of all assemblies to generate an integrated set of contigs?

In this study, state-of-the-art assemblers were used to generate different sets of contigs for bacterial genomes. We have developed and built a Contig Integrator for Sequence Assembly (CISA) to integrate the sets of contigs from different assemblers, and have evaluated the quality of our integrated assembly. Compared with the assemblies generated by each individual assembler and assembly integrator, the hybrid assembly integrated by CISA exhibits superior contiguity and accuracy.

## Materials and Methods

### Algorithms

The contig integrator consists of four major phases: (1) identification of the representative contigs and possible extensions (2) removal and splitting of the contigs that may be misassembled, and clipping of uncertain regions that are located at the extremities of the contigs, (3) iterative merging of the contigs with a minimum 30% overlap and estimating the maximal size of repetitive regions, and (4) merging of the contigs based on the size of repetitive regions. We employed NUCmer [9] and blastn with the default settings to implement sequence alignments for phases (1) and (2), and phases (3) and (4), respectively.

**Figure 1:**
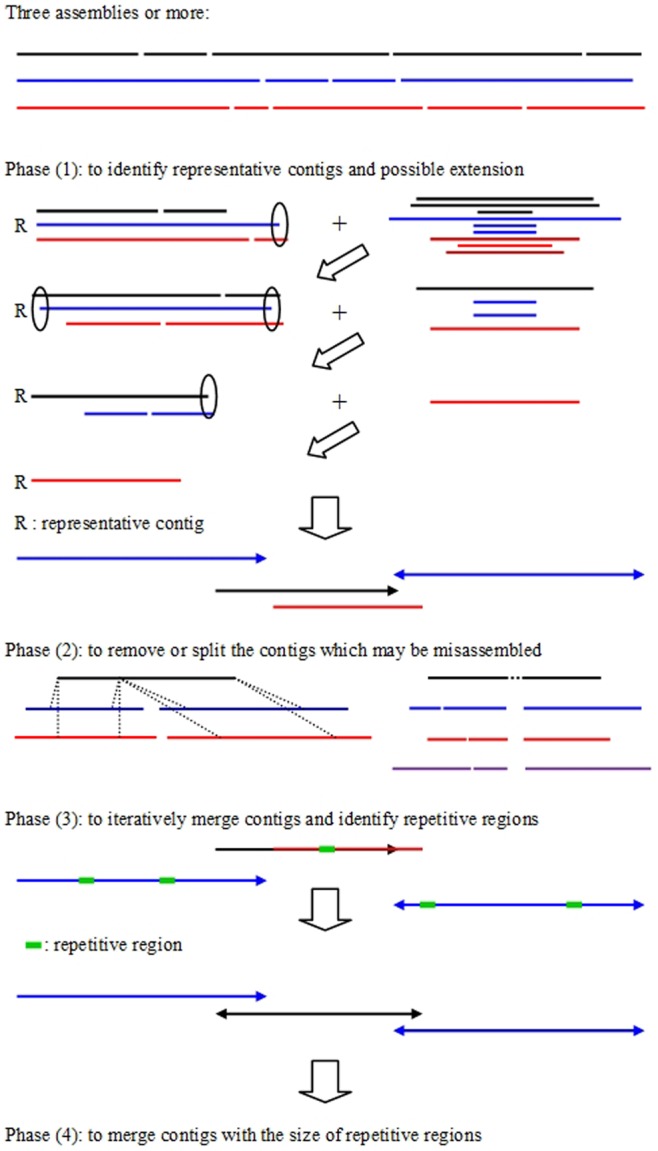
A schematic overview of CISA. Phase (1): employing the largest contig as a representative contig and identifying the contigs which were aligned to the ends of the representative contig with more than 80% alignment to perform possible extension (hollow ellipses and solid arrows represent before and after extension, respectively). Phase (2): removing and splitting misassembled contigs. Two misassembled contigs are shown in black. The left element represents a misjoined error because it was assembled in two different contigs in two individual assemblies (blue and red); the right element represents an insertion error (dot), which was never seen in other assemblies. Phase (3): iteratively merging contigs with a proper end-to-end overlap and estimating the size of repetitive regions. Phase (4): merging two contigs with a small overlap greater than the maximum size of repetitive regions.

A schematic overview of CISA is displayed in Fig. 1. In phase (1), we used the largest contig as a representative contig and removed the contigs whose major portion of sequence (>95% alignment rate and >95% sequence identity) can be found in the representative contig from the query set. Additionally, contigs aligned to the ends of the representative contig with more than 80% alignment (>95% sequence identity) were used for possible extension (hollow ellipses and solid arrows represent before and after extension, respectively). The largest contigs of the remaining query set (in the right-hand side) were iteratively selected as subsequent representative contigs. In phase (2), each representative contig was examined for misassembly and uncertainty of ends or gaps based on the sequence alignments to all contigs in the query set. If a representative contig (e.g., the black contig in the left-hand side) was found in the middle of two individual contigs in a single assembly, suggesting misassembly of the contig, the representative contig was removed and a supplemental representative contig was introduced. If a representative contig contained a region in which no other contigs could be aligned, e.g., the black contig in the right-hand side, the uncertain region was clipped and partitioned accordingly. In the later two phases (3) and (4), we aimed to merge contigs with overlaps. If two contigs comprised of a proper end-to-end overlap (with the aligned region >30% of the smaller of the lengths of the two contigs), they were merged. Since a contig may bridge two contigs, the merge processes were performed iteratively until there was no sequence alignment with over 30% alignment rate. In order to define repetitive regions, we examined the sequence alignments in phase (3) and identified an identical region in a contig, which was aligned to at least two different regions. The maximum size of the repetitive regions was therefore obtained, and with that, in phase (4), two contigs were merged with a small overlap (greater than the maximum size of repetitive regions) while preventing misassembly.

In order to quantify the accuracy of the integrated contigs, several bacterial genomes were employed, which have been published with their complete reference genomes and available sequenced reads. Each of the integrated contigs generated in this study was evaluated by Mauve assembly metrics [8]. Given a reference genome and an assembled genome, the following metrics are assessed with Mauve assembly metrics: the double cut and join (DCJ) distance, the percentages of extra and missing bases (%Extra and %Missed), the number of intact coding sequences based on the location of substitution and frame-shift errors between the assembly and reference, and the maximum length of contig. Moreover, we calculated contig N50s [10] and used all CDS in the reference genome to blast the contigs and thus defined a blast-based intact CDS for which the best match could be identified (>99% alignment rate) from the integrated contigs with a sequence identity over 99%. The correctness of the assemblies was also assessed by counting the assembly errors including the indels longer than 5 bases (indel> = 5) and two types of misjoin (inversion, relocation) [11].

### Materials

With respect to *Escherichia coli* K12 MG1655 (*E. coli*), we began with sequence reads of 36 bp deposited in the NCBI Sequence Read Archive (SRA), which were sequenced from the Illumina paired-end library corresponding to an insert size of 200 bp (SRR001665). 100 bp pair-end sequencing data were also downloaded from Illumina. For *Staphylococcus aureus,* two strain MW2 (*S. aureus* 35 bp) and subsp. *aureus* USA300_TCH1516 (*S. aureus* 101 bp) were downloaded from the supplemental data of D. Hernandez *et al.* [12] and S. L. Salzberg *et al*. [11], respectively. Those datasets were employed for the *de novo* sequence assembly by various assemblers including Abyss [13], CLC, Edena [12], SOAPdenovo [14], and Velvet [15]. Sequence contigs obtained from the assemblers were subsequently input into CISA to generate an integrated set of contigs. Since CISA is intended to serve as a contig integrator, its input is a set of contigs from at least three different assemblies. We directly used the assemblies of the *Haloferax volcanni* strain DS2 (*H. volcanni*) to evaluate the performance of CISA. The different sets of contigs for *H. volcanni* are available from A. E. Darling *et al.* [8]. The results obtained from CISA have been compared with those obtained from different contig integrators including GAA [3], MAIA [5], and minimus2 [6].

## Results and Discussion

**Figure 2:**
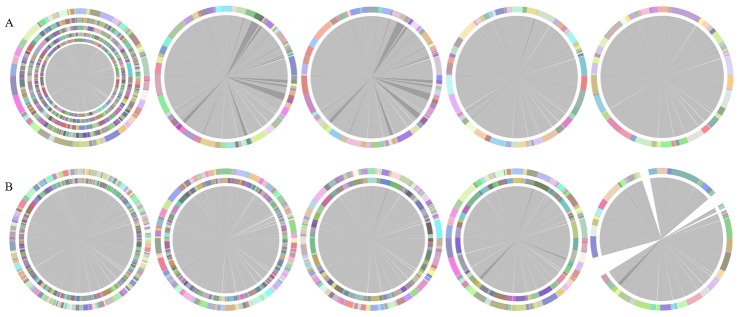
Integration of five assemblies of 36 bp paired-end reads for *E. coli* using CISA and minimus2. (A) From left to right, CISA integrates the five assemblies (Abyss, CLC, Edena, SOAPdenovo and Velvet from the outer to the inner) and sequentially generates the processed contigs after phase (1), (2), (3), and (4). Each contig color is randomly assigned. The white and grey segments in the inner circle show missing and laying of contigs in the genome. The dark-grey segments represent overlaps between contigs. (B) From left to right, minimus2 firstly merges SOAPdenovo (the inner) with CLC (the outer), then merges the output (the inner) with Velvet (the outer) in the second run, merges the output (the inner) with Edena (the outer) in the third run, and finally merges the output (the inner) with Abyss (the outer) in the fourth run to generate a hybrid assembly.

CISA is implemented in Python and requires MUMmer and BLAST+ installed on the local machine. A user supplies a set of contigs from at least three assemblers in FASTA format to CISA to obtain integrated contigs. The performance of CISA was compared with the assemblies generated by various assemblers and with the hybrid contigs merged by GAA, MAIA and minimus2 on all datasets. The overview of each set of contigs for *E. coli*, *S. aureus*, and *H. volcanni* evaluated by Mauve assembly metrics [8], contig N50 [10], blast-based intact CDS, and assembly errors [11] with their reference genomes are shown in Table 1, 2, 3, respectively. These examples, including the *de novo* assemblies yielded from various state-of-the-art assemblers with Illumina single-end and paired-end sequencing reads of *S. aureus* and *E. coli*, as well as the hybrid assemblies for *H. volcanni*, were successfully integrated by CISA. As shown in Table 1 and Table 2, CISA effectively reduces the number of contigs, the DCJ distance, and the percentage of missing bases, and increases the contig N50, the maximum length of contigs, and the intact CDS in comparison with various state-of-the-art assemblers. Obviously, with the four sequential phases of CISA, the five assemblies (in the case of 36 bp paired-end reads for *E. coli*) were integrated into the improved set of contigs (Fig. 2A), providing the smallest number of contigs while achieving the most intact CDS. As for the case of *H. volcanni*, CISA is able to integrate the three assemblies obtained from different sequencing platforms into a set of contigs, which exhibits increased contiguity (i.e., a reduction in the number of contigs from a range of 157 – 1555 to 72) and comparable accuracy to the three assemblies (Table 2). CISA was designed to take the advantages of various assemblies and therefore enables improved genome assembly, which may expedite in completing a genome.

**Table 1:** Evaluation of sequence assemblies for *E. coli.*

Name		Num Contigs	Assembly Bases	DCJ Distance	%Missed	%Extra	Intact CDS	Max Contig	N50	Blast-based Intact CDS	Assembly errors (Indel> = 5, Inversion, Relocation)
*Escherichia coli* K12 MG1655 (genome size = 4639675, number of CDS = 4320) with 36 bp paired-end reads											
Abyss		133	4626205	107	1.25	0.64	4263	222425	96511	4249	8 (6, 0, 2)
CLC		379	4546926	304	2.81	0.07	4258	107342	29905	4228	2 (0, 1, 1)
Edena		211	4569446	154	1.87	0.05	4254	186686	57790	4191	4 (2, 1, 1)
SOAPdenovo		553	4547211	475	2.68	0.15	4220	103369	17944	4131	1 (0, 0, 1)
Velvet		283	4550675	207	2.51	0.06	4246	166094	54359	4194	8 (5, 0, 3)
**CISA**		**72**	**4627549**	**70**	**1.07**	**0.69**	**4276**	**312018**	**126254**	**4276**	**11 (8, 0, 3)**
GAA#		314	4578451	245	2.09	0.23	4248	157184	51218	4205	4 (2, 0, 2)
GAA*		311	4602917	224	2.01	0.26	4244	163308	51085	4208	5 (3, 0, 2)
MAIA		110	4513348	96	2.80	0.02	4272	312145	126075	4212	5 (2, 0, 3)
minimus2#		155	4598769	133	1.94	0.71	4262	202745	86241	4243	8 (5, 1, 2)
minimus2^A^		74	4608653	68	1.66	0.77	4270	417704	134584	4262	10 (7, 1, 2)
minimus2^B^		69	4215087	69	11.82	2.68	4269	312145	126233	3855	10 (5, 2, 3)
minimus2*		73	4597392	67	3.36	2.25	4268	296685	129557	4199	11 (7, 1, 3)
*Escherichia coli* K12 MG1655 (genome size = 4639675, number of CDS = 4320) with 100 bp paired-end reads											
Abyss		190	4642915	119	1.08	0.51	4244	222412	68544	4243	3 (0, 0, 3)
Edena		421	4584984	331	1.87	0.15	4175	104739	24632	4048	3 (1, 1, 1)
SOAPdenovo		560	4596003	272	2.41	0.14	4209	105615	31837	4105	3 (1, 0, 2)
Velvet		264	4569720	191	2.22	0.09	4228	161713	47550	4159	16 (4, 7, 5)
**CISA**		**110**	**4641820**	**106**	**0.96**	**0.84**	**4251**	**222524**	**79212**	**4248**	**12 (3, 4, 5)**
GAA#		354	4616496	223	1.63	0.30	4218	153242	46435	4152	6 (2, 1, 3)
GAA*		311	4636486	222	1.28	0.49	4213	162326	50871	4152	6 (3, 1, 2)
MAIA		263	4351338	239	9.10	0.48	4226	161713	47928	3882	1 (0, 0, 1)
minimus2#		164	4593120	141	2.49	0.76	4241	184547	63799	4177	10 (4, 3, 3)
minimus2*		94	4588207	88	6.95	4.52	4244	225809	88309	4077	13 (6, 3, 4)

# lease note that GAA and minimus2 were designed to merge two assemblies at a time. All 2-combinations were thus performed and the average scores were taken.

* Please note that GAA and minimus2 were performed iteratively in ten random orders and the obtained scores were averaged.

A: The order of combination is Abyss + CLC + Edena + SOAPdenovo + Velvet

B: The order of combination is SOAPdenovo + CLC + Velvet + Edena + Abyss

**Table 2:** Evaluation of sequence assemblies for *S. aureus.*

Name		Num Contigs	Assembly Bases	DCJ Distance	%Missed	%Extra	Intact CDS	Max Contig	N50	Blast-based Intact CDS	Assembly errors (Indel> = 5, Inversion, Relocation)	
*Staphylococus aureus* strain MW2 (genome size = 2820462, number of CDS = 2651) with 35 bp reads												
ABySS		929	2769174	898	3.36	0.31	2480	32717	7810	2337		2 (0, 0, 2)
Edena		931	2757686	882	3.49	0.25	2463	37100	6969	2293		2 (0, 0, 2)
SOAPdenovo		944	2781524	917	2.80	0.42	2485	26967	6427	2348		3 (0, 0, 3)
Velvet		1152	2775301	1124	3.14	0.54	2421	22892	5348	2238		2 (0, 0, 2)
**CISA**		**665**	**2776108**	**635**	**2.81**	**0.20**	**2551**	**42008**	**10605**	**2446**		**2 (0, 0, 2)**
GAA#		1015	2783335	959	2.90	0.45	2464	29922	6634	2312		2 (0, 0, 2)
GAA*		1046	2794625	956	2.77	0.53	2463	30079	6750	2314		2 (0, 0, 2)
MAIA		769	2776022	767	3.66	0.69	2474	51874	8610	2360		1 (0, 0, 1)
minimus2#		739	2770378	723	3.09	0.32	2516	35867	9006	2401		2 (0, 0, 2)
minimus2*		568	2769000	560	3.02	0.26	2548	42022	11094	2450		2 (0, 0, 2)
*Staphylococus aureus* strain subsp. *aureus* USA300_TCH1516 (genome size = 2903081, number of CDS = 2693) with 101 bp paired-end reads												
ABySS		659	2854631	590	2.41	0.25	2486	35459	9229	2305		7 (2, 0, 5)
Edena		3287	2557545	3143	13.46	0.62	1909	8680	1256	1053		4 (1, 0, 3)
SOAPdenovo		674	2872327	522	2.45	0.36	2539	47607	9762	2361		3 (2, 0, 1)
Velvet		502	2858949	432	2.39	0.27	2556	54726	13005	2421		19 (10, 4, 5)
**CISA**		**347**	**2866024**	**330**	**2.08**	**0.40**	**2572**	**54747**	**15327**	**2482**		**20 (6, 5, 9)**
GAA#		1287	2798306	1166	4.96	0.47	2371	36637	8374	2045		9 (4, 1, 4)
GAA*		1150	2827068	1022	4.25	0.62	2401	38358	9026	2123		10 (4, 2, 4)
MAIA		505	2859291	498	3.57	0.86	2552	52790	12800	2376		2 (1, 0, 1)
minimus2#		421	2863142	399	2.42	0.54	2552	50951	13159	2425		13 (5, 2, 6)
minimus2*		302	2852733	302	3.12	0.92	2579	54766	16835	2468		22 (6, 7, 9)

# Please note that GAA and minimus2 were designed to merge two assemblies at a time. All 2-combinations were thus performed and the average scores were taken.

* Please note that GAA and minimus2 were performed iteratively in ten random orders and the obtained scores were averaged.

**Table 3:** Evaluation of sequence assemblies for *H. volcanni.*

Name		Num Contigs	Assembly Bases	DCJ Distance	%Missed	%Extra	Intact CDS	Max Contig	N50	Blast-based Intact CDS	Assembly errors (Indel> = 5, Inversion, Relocation)
*Haloferax volcanni* strain DS2 (genome size = 4012900, number of CDS = 4015)											
assembly1		157	3920004	117	2.94	0.03	3981	217295	127504	3953	8 (3, 0 5)
assembly2		1555	3855484	1674	4.93	0.43	3557	55518	9092	3144	201 (154, 1, 46)
assembly3		580	3871717	602	4.05	0.51	3575	53121	12830	3411	499 (479, 9, 11)
**CISA**		**72**	**4041406**	**75**	**4.90**	**3.08**	**3960**	**222325**	**109517**	**3910**	**38 (31, 3, 4)**
GAA#		693	3934772	688	3.94	1.37	3730	122155	54582	3593	237 (213, 3, 21)
MAIA		893	3619301	875	24.19	6.95	3671	265643	16602	2946	59 (56, 0, 3)
minimus2#		179	4168210	192	5.35	8.03	3754	224742	113003	3855	212 (200, 4, 8)
minimus2*		71	4195273	77	4.46	7.87	3796	332251	167497	3951	186 (177, 4, 5)

# Please note that GAA and minimus2 were designed to merge two assemblies at a time. All 2-combinations were thus performed and the average scores were taken.

* Please note that GAA and minimus2 were performed iteratively in ten random orders and the obtained scores were averaged.

GAA, a graph accordance assembly program [3], was firstly employed to merge two assemblies of *E. coli* with 36 bp paired-end reads. Edena was input as the query assembly to improve the target assembly from Abyss using GAA. However, in comparison with the assembly yielded by Abyss, minor improvements in the missed and extra rates were obtained (from 1.25 to 1.18, and from 0.64 to 0.62, respectively; the details are furnished on the CISA website). Furthermore, 20 varied combinations (implementing either two out of the five assemblies) were performed with GAA and then the evaluated scores were averaged. The results (GAA^#^), in contrast to the state-of-the-art assemblers, indicate that GAA did not significantly enhance the results. Considering an equal number of utilized assemblies for the sake of comparison, we began with one set of two assemblies and iteratively added an additional assembly to the GAA-generated contigs. Of the 120 possible permutations, ten sets were randomly selected as inputs to GAA; average scores (GAA*) were obtained, which reveal that the increased number of assemblies serves to enhance the performance in a minimal fashion.

MAIA, an algorithm that integrates multiple genome assemblies, was also studied [5]. However, a relatively closely-related reference genome is required for implementation in this algorithm. The MAIA protocol was strictly followed in order to integrate the five assemblies in the case of *E. coli* with a related genome sequence, *E coli* str. K-12 substr. DH10B. Unfortunately, MAIA failed to complete the integration and error messages were accordingly displayed. The reference genome MG1655 was subsequently applied to MAIA for the integration of the assemblies and a single contig was yielded with 126761 uncalled bases (i.e., Ns). The integrated contig was evaluated with Mauve assembly metrics, however an error was encountered. The assembly was, therefore, partitioned into contigs (split at >10 Ns) and evaluated. In all three examples, the performance of MAIA was inferior to CISA, even with the aid of the reference genome (Table 1, 2, 3).

Minimus2, a modified version of the Minimus pipeline implemented for merging two sequence sets, has been widely utilized for the hybrid merger of two assemblies originating from various sequencing technologies (e.g. Illumina and 454) [6]. In light of the rapid development of assemblers, one could acquire a number of assemblies by executing various assemblers. However, a conundrum arises under this scenario as the user must select two specific sets from the substantial body of assemblies. In accordance with the strategy that was employed with GAA previously, a 2-fold combination evaluation was performed leveraging minimus2 to obtain the averaged scores (denoted as minimus2^#^ in Table 1, 2, 3). The results reveal that minimus2 could improve the accuracy and contiguity of the single assemblies and outperform GAA and MAIA alone. Minimus2 was further applied to merge multiple assemblies in an iterative manner, e.g., two different orders of the five-fold combination of assemblies: Abyss + CLC + Edena + SOAPdenovo + Velvet and SOAPdenovo + CLC + Velvet + Edena + Abyss, as illustrated in minimus2^A^ and minimus2^B^, respectively, in Table 1. As can be interpreted from minimus2^A^, minimus2 is likely to enhance the number of contigs, intact CDS, and the size of N50. However, minimus2 exhibits insufficient ability to merge the assemblies in the order S+C+V+E+A, inferred from exceeding a 10 percent of genome loss performance criteria in the final merging iteration (the hybrid assembly is provided in Fig. 2B). Such behavior can be ascribed to the irregular disposal of ambiguous contigs when merging the output of S+C+V+E with the assembly of Abyss (in the fourth run, Fig. 2B); these discarded contigs comprised the missed genome. Furthermore, it is crucial to note that the number of intact CDS reported by Mauve assembly metrics for minimus2^B^ was clearly overestimated owing to conflicting results yielded by this algorithm and the high rate of missing contigs. Although it was found that minimus2 can improve contiguity by reducing the number of contigs via an average scoring of ten randomly selected hybrid assemblies (minimus2*), the algorithm lost accuracy in generating the larger missed and extra rates as well as the smaller number of intact CDS than CISA.

In the case of *H. volcanni*, the numbers of intact CDS estimated by Mauve assembly metrics and blast were not consistent. We assume that the number of intact CDS of Mauve assembly metrics was estimated by examining frame-shift errors; this approach is thus not consistent with the blast-based intact CDS. A CG-pipeline [16] was thus employed, which implements GeneMarkS and Glimmer3 to predict features for the integrated contigs generated by CISA and minimus2 (in the merging order of assembly1 + assembly2 + assembly3). Following feature predictions, blast was performed for the features predicted from the contigs integrated separately by CISA and minimus2 against all CDS in *H. volcanni*. The numbers of intact CDS generated by CISA and minimus2 were 3822 and 3783, respectively, which suggests that less frame-shift errors (i.e., assembly errors in indels) are generated while using CISA.

Note that the quality of an assembly was generally evaluated with the number of contigs or N50 owing to the unavailability of reference genomes, in practice. One should, therefore, exercise caution while executing minimus2, as the quality of the assembly is dependent upon the merging order. In contrast, the low rate of missing and extra bases as well as the large number of intact CDS of the assemblies substantiates that CISA facilitates the integration of a high-fidelity assembly, and, by virtue of its aforementioned properties, CISA has been demonstrated as a favorable utility to integrate multiple assemblies and generate an integrated set of contigs for bacterial genomes.
